# Area-Selective Atomic Layer Deposition for Next-Generation Semiconductor Processing: Mechanisms of Selectivity Breakdown and Process Integration Strategies

**DOI:** 10.3390/mi17101152

**Published:** 2026-09-30

**Authors:** Yuna Park, Sooyeon Kim, Jinsoo Shin, Eunmi Park, Sung Gyu Pyo

**Affiliations:** School of Integrative Engineering, Chung-Ang University, Seoul 06974, Republic of Korealeafgreen0504@cau.ac.kr (J.S.); eunmi5308@cau.ac.kr (E.P.)

**Keywords:** area-selective atomic layer deposition, selectivity breakdown, nucleation, inhibitor, atomic layer etching, self-aligned patterning, semiconductor process integration

## Abstract

As device miniaturization and three-dimensional integration progress, thin-film processes for semiconductor devices increasingly require atomic-level control not only of thickness and composition but also of growth location. Area-selective atomic layer deposition (AS-ALD) is a bottom-up process that exploits differences in surface reactivity between growth and non-growth areas to form thin films only at the intended locations. Because it enables self-aligned patterning and can reduce subsequent etching steps, AS-ALD has drawn attention as a candidate technology for next-generation semiconductor manufacturing. However, the high initial selectivity obtained on planar substrates is not always retained on real patterned or high-aspect-ratio structures. This review examines the process applicability of AS-ALD based on studies published through 2026, focusing on the selectivity window, the maximum selectively deposited thickness, the nucleation density in the non-growth area, and the preservation of pattern shape. Across the literature, the turning point of selectivity breakdown converges on the moment at which the first stable nucleus forms in the non-growth area. Residual surface defects and pinholes in the inhibition layer, together with precursor physisorption, penetration, thermal degradation, and lateral diffusion, all act to bring this transition forward in time. Molecular design strategies (inherent selectivity, self-assembled monolayers, small-molecule inhibitors, surface pretreatment, and combined precursor–inhibitor design) and process control strategies (inhibitor regeneration, ALD/ALE supercycles, and reactor control) are compared according to which stage of the nucleation pathway they intervene in. Pattern- or device-level demonstrations have accumulated for back-end-of-line interconnects and resistive random-access memory, whereas for DRAM, 3D NAND, and gate-all-around transistors most of the evidence remains at the level of material and process concepts. The performance of AS-ALD is difficult to judge from the initial selectivity value alone; a more direct criterion is whether the selectivity window—defined with respect to the target thickness and the tolerable defect level of the actual device structure—can be secured.

## 1. Introduction

The control targets required in semiconductor thin-film processes have expanded beyond thickness to include growth location, interfaces, composition, and defects. Physical vapor deposition (PVD) offers a high deposition rate and is favorable for forming metal films, but its strongly directional incidence limits uniform coverage of deep trenches and channel holes. Chemical vapor deposition (CVD) uses gas-phase and surface reactions to achieve relatively high throughput, but because reactants are supplied simultaneously, it can suffer from overgrowth near the inlet, gas-phase nucleation, and concentration gradients inside high-aspect-ratio structures. Atomic layer deposition (ALD) temporally separates the precursor and co-reactant and drives each reaction to surface saturation, thereby providing cycle-by-cycle thickness control and excellent step coverage [1,2,3,4]. While this property makes ALD well suited to blanket deposition that uniformly covers exposed reactive surfaces, structures that require localized film formation still need additional lithography and etching steps.

AS-ALD complements the limitations of blanket deposition by defining the growth location through surface chemistry. In the growth area (GA), precursor adsorption and nucleation are promoted, whereas in the non-growth area (NGA), reactive sites are removed or an inhibitor is introduced to delay initial nucleation. Because an already-formed material boundary serves as the alignment reference, self-aligned structures can be realized without a separate overlay step [5,6,7,8,9]. However, observing selective deposition itself does not necessarily imply process applicability. If a stable nucleus forms in the NGA before the target thickness is reached, subsequent ALD reactions proceed on that surface and non-selective growth expands rapidly.

Existing reviews have broadly summarized the implementation approaches and application areas of AS-ALD [5,6,7,8,9]. Recent studies indicate that the factors governing selectivity are not limited to a simple difference in surface reactivity. Precursor physisorption and penetration into the inhibition layer, surface diffusion, reactant residence time, and even three-dimensional topography jointly govern nucleation behavior. This article focuses on this shift, drawing on literature published through 2026. It also reviews approaches that are difficult to describe using the traditional GA/NGA distinction alone, such as two-dimensional lateral superlattices, local activation of chemically homogeneous surfaces, topographically selective deposition, and inhibitor-free processes [10,11,12,13,14,15,16]. When comparing across studies, priority is given to the thickness over which selectivity is maintained, pattern-size dependence, defect density, and tool-level reproducibility, rather than to the initial selectivity value alone.

**Figure 1:**
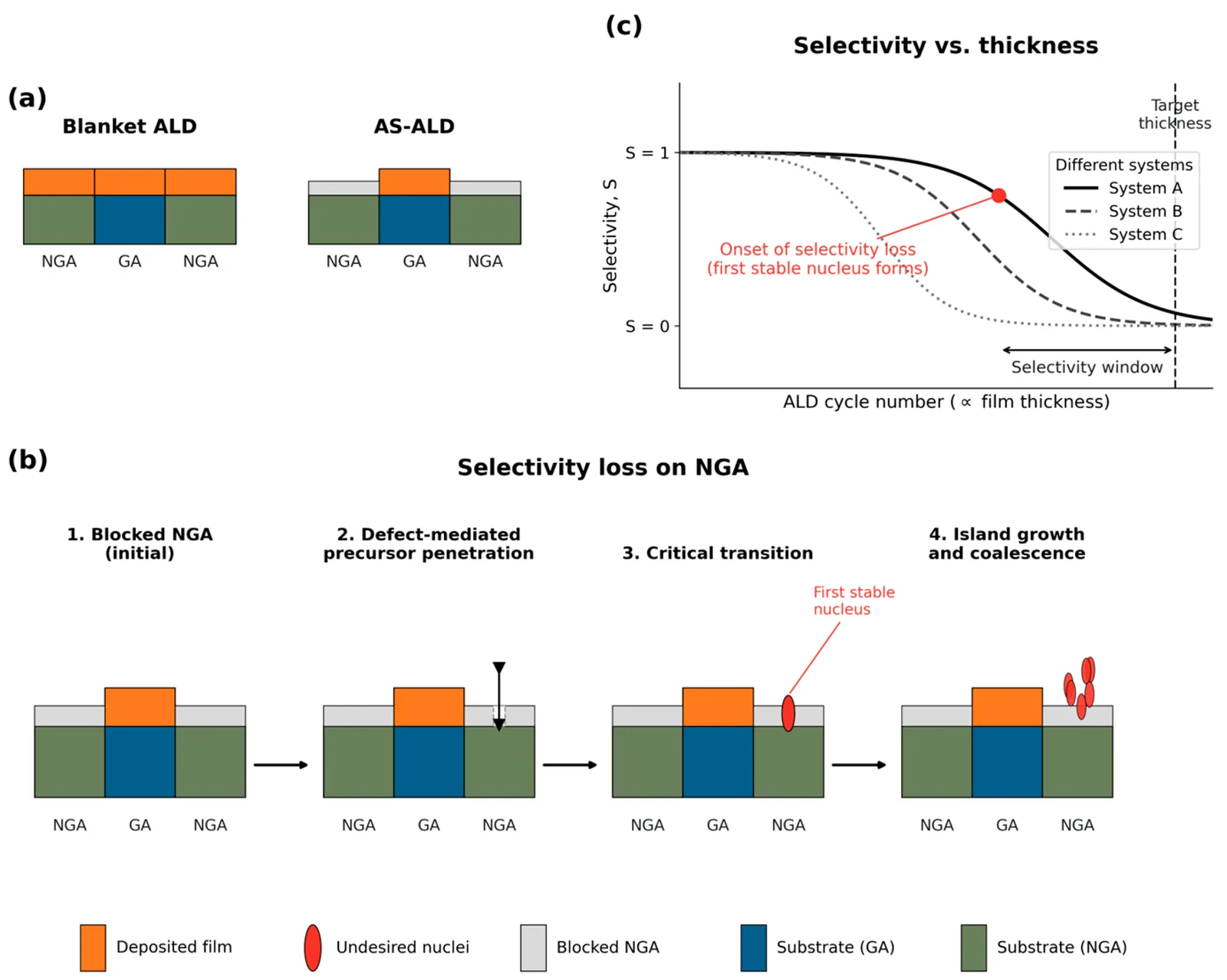
Concept of AS-ALD and selectivity breakdown. (**a**) Difference between blanket ALD and AS-ALD on the same patterned substrate; (**b**) the process of selectivity breakdown in the NGA: initial blocking state, defect-mediated precursor penetration, formation of the first stable nucleus, and island growth and coalescence; (**c**) schematic of selectivity as a function of ALD cycle number (or film thickness) and the selectivity window.

The following sections first address the formation and breakdown of selectivity, centered on the process by which a stable nucleus forms in the NGA. Next, the effects of molecular design, inhibitor-layer regeneration, ALD/ALE defect correction, and reactor control on the selectivity window are examined. The device-application section distinguishes the application sites and demonstration levels for back-end-of-line interconnects, memory, and transistors, and the final section discusses the verification items required for transition to manufacturing. The core concepts and logical flow of this review are summarized in Figure 1.

## 2. Formation and Breakdown of Selectivity: Formation of the First Stable Nucleus in the Non-Growth Area

The selectivity of AS-ALD can be expressed as the difference in the amount of material formed in the GA and the NGA. When defined in terms of growth rate or film thickness, selectivity S is given by Equation (1) [5,7].

*S* = (*G_GA_* − *G_NGA_*)/(*G_GA_* + *G_NGA_*) (1)

In the ideal case, growth in the NGA approaches zero and S approaches 1. However, a high selectivity calculated from an average film thickness does not guarantee a defect-free surface. Sparse, island-like nuclei in the NGA are present at a level that is difficult to quantify, rather than truly undetectable, by average-thickness measurements. In subsequent ALD cycles, these nuclei become new growth surfaces that rapidly amplify non-selective growth.

Selectivity evaluation should not be limited to the initial S value. The selectivity window, the nucleation delay, the maximum selectively deposited thickness, the nucleus density in the NGA, and the shape preservation at pattern boundaries must all be examined together. Among these, the event that determines selectivity breakdown is the moment at which the first stable nucleus forms in the NGA. Here, a “stable” nucleus is understood, in the sense of classical nucleation theory, as an adsorbed cluster that has exceeded the critical size above which further growth lowers the free energy of the system, so that continued growth becomes more probable than dissolution. This critical size is not a fixed atom count or diameter; it depends on the supersaturation and on the precursor–substrate and precursor–precursor interaction energies of the specific material system, so a nucleus considered “stable” in one AS-ALD process can correspond to a different physical size than in another. Surface defects and pinholes in the inhibition layer, precursor physisorption and penetration, thermal degradation, and mass transport near boundaries all act to bring this moment forward.

This is also why results obtained on planar substrates are difficult to apply directly to real patterns. Actual structures combine line-edge roughness, corners, and sidewalls with etch residues and differing surface termination states. At these locations, inhibitor arrangement and precursor delivery differ from those on flat regions. It is therefore more accurate to regard selectivity not as a fixed property of a given material combination but as a process characteristic that varies with surface state, reactant residence time, topography, and cycle number.

When comparing selectivity values across studies, it is necessary to check both the measured quantity and the detection limit. Average thickness measurements easily miss the small number of nuclei formed over a wide NGA, and XPS and ellipsometry differ in analysis depth and spatial resolution. In particular, comparing raw ellipsometric psi/delta spectra or wavelength-dependent shifts can reveal that a surface has changed even before a validated optical model exists for the sparse, non-uniform nucleation layer; such early-stage changes are therefore better described as not reliably quantifiable rather than as undetectable [15]. Parsons proposed a quantitative model that separates the initial nucleation delay from the difference in growth rate [17]. Claessens et al. used Rutherford backscattering spectrometry to distinguish the areal density of Ru on the top and sidewalls of SiO_2_/TiN patterns with a 35 nm line width and 90 nm pitch [18]. By achieving a detection limit of roughly 10^13^ atoms cm^2^, they were able to quantify local deposition at a stage before it becomes apparent in the average thickness. More recently, computational fluid dynamics models that combine reactor flow with surface reaction kinetics, and machine-learning searches using precursor molecular descriptors, have been proposed [19,20]. Selectivity evaluation is thus broadening from a comparison of final thicknesses to an analysis of position-dependent mass transport and nucleation probability.

### 2.1. Initial Adsorption and Nucleation: Scope and Limits of Inherent Selectivity

The initial growth behavior of ALD depends on surface hydroxyl and hydrogen termination, amine groups and oxide defects, and the catalytic character of metals [2,3,4]. Inherent selectivity exploits this reactivity difference without a separate inhibition layer. Selective deposition of metal oxides using oxygen activated on noble-metal surfaces [21] and the redox-coupled reaction at the Ta-oxide/Cu-SiO_2_ interface [22] show that surface chemistry itself can determine the growth location. This approach has the advantage that no organic inhibitor remains, but as cycles accumulate, adsorption and nucleation eventually begin in the NGA as well, which is why the thickness over which selectivity is maintained tends to be limited.

The influence of surface termination is also evident in nucleation studies of conventional ALD. In a 100 °C TiCl_4_/H_2_O process, hydroxyl-rich SiO_2_ showed a higher initial TiO_2_ growth rate than hydrogen-terminated Si, and the growth rate on both surfaces changed over the first several cycles [23]. In Ru ALD, the initial nucleus density and island coalescence behavior determined the continuity of the final film [24]; treating the surface with small organometallic molecules changed not only the number of nucleation sites but also surface diffusion and particle coalescence [24]. In O_3_-based metal-oxide ALD, reactive oxygen accumulated on the surface alters the reactivity of the subsequent precursor pulse [25], and in comparisons of Al precursors, the degree of ligand chlorination and the alkyl structure affected the reaction pathway and byproduct removal [26]. The difference between GA and NGA thus originates less from a fixed substrate property than from the difference in surface state formed after each half-cycle.

Initial nucleation cannot be explained by chemisorption alone. Seo et al. showed that, on an ODPA-passivated surface, TMA can physisorb even without directly reacting with the methyl-terminated layer [27]. The species remaining on the surface were converted into nuclei during the subsequent H_2_O exposure. By adjusting the TMA partial pressure and purge conditions, the selective growth window of Al_2_O_3_ was extended to about 370 cycles, or 60 nm. Selectivity is thus sensitive not only to surface reactivity but also to the adsorption–desorption equilibrium and the rate of removal of residual reactants.

**Figure 2:**
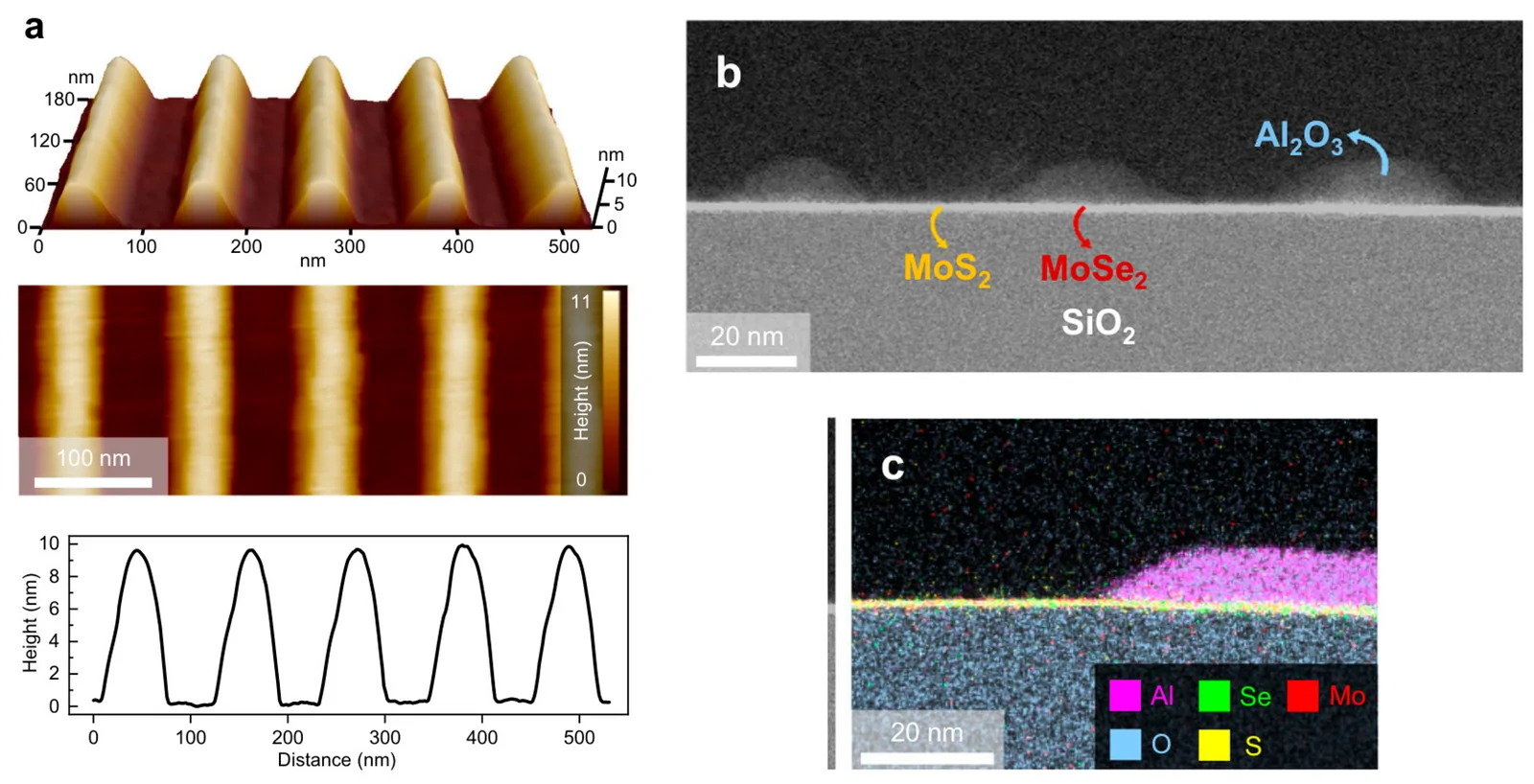
Selective Al_2_O_3_ deposition on a MoS_2_-MoSe_2_ lateral superlattice. (**a**) Three-dimensional AFM topography, planar morphology, and height profile; (**b**) cross-sectional HAADF-STEM image; (**c**) cross-sectional EDS elemental distribution. Adapted and reconstructed from Park et al. [11], Figure 2a–c (CC BY 4.0).

Surface diffusion is another factor that changes the spatial distribution of selectivity. Klement et al. showed that varying the diffusion length changes the pattern of selective growth [28], and Grillo et al. reported that area-dependent differences in surface diffusion alter the distribution of nuclei in Ru ALD [29]. A recent study using a two-dimensional monolayer lateral superlattice also formed selective patterns on the order of 10 nm through surface-dependent differences in adsorption and diffusion [11]. Inherent selectivity can therefore be understood less as an absolute distinction between GA and NGA and more as a kinetic window during which the difference in adsorption, desorption, and diffusion rates between the two surfaces persists. Representative morphological and compositional analysis results are shown in Figure 2.

### 2.2. Formation and Breakdown of Inhibition Layers: Self-Assembled Monolayers and Small-Molecule Inhibitors

A self-assembled monolayer (SAM) consists of a head group that binds to the substrate and an alkyl or fluorinated tail that blocks precursor access. Early studies on HfO_2_ AS-ALD showed that alkylsilane and phosphonic-acid-based SAMs provide a clear nucleation delay [30,31]. Later results revealed that the blocking performance is not determined by the molecular species alone; the bonding state to the substrate and the packing density of the molecular arrangement are also important. Dodecanethiol formed a more stable arrangement on partially oxidized Cu than on metallic Cu, and when combined with an unsaturated precursor supply condition, a selectivity of 0.99 was achieved for 4 nm of Al_2_O_3_ [32,33,34,35]. Excessive oxidation, however, disrupted uniform SAM formation and lowered selectivity again.

Research interest in SAMs has shifted from whether they provide inhibition at all to how faithfully the chemical information written on a surface can be transferred into an actual film pattern. Early studies that controlled the growth location of HfO_2_ on chemically patterned substrates and extended this to positive pattern transfer [36,37] demonstrated that SAMs function both as a passivation layer and as a carrier of pattern information. This was followed by reports of a thermally stable Ge/Si monolayer resist [38], spatially selective ALD using microcontact-printed resists together with Pt deposition on YSZ [39,40], and a water-soluble polymer resist [41], broadening the range of patterning approaches and deposited materials. The key variable here is less the identity of a specific inhibitor than the sharpness of the surface-modification boundary and its stability during processing. Nevertheless, many studies remain confined to micrometer-scale planar patterns; statistics of molecular-scale defects, boundary roughness, and lateral growth in sub-10 nm structures and three-dimensional topographies still require separate verification.

When chemically similar oxides must be distinguished, the role of surface-selective bonding becomes more important. A study exploiting the different adsorption behavior of ODPA on Al_2_O_3_ and HfO_2_ reported a selectivity above 0.9 for about 14 nm of ZnO and 2.5 nm of Al_2_O_3_ [42]. Bulk composition alone is a poor predictor of selectivity on oxides; surface hydroxyl density, solvent, pretreatment history, and inhibitor arrangement must all be considered together.

The blocking performance of a SAM can be separated into the quality of the initial barrier and the survivability of that barrier during ALD cycling. Substrate-dependent orientation and order of alkylphosphonic acids, and the low surface energy of fluorinated SAMs, determine the initial barrier against precursor access [43,44]. A study that tracked the breakdown of alkanethiol SAMs found that local defects were converted into nuclei with increasing cycle number even while the average coverage was maintained [45]—evidence that a dense monolayer alone cannot guarantee a long selectivity lifetime. A process that simultaneously promoted GA nucleation while blocking the NGA actively widened the growth-rate difference between the two regions [46]. Temperature-dependent vibrational spectroscopy identified thermally induced gauche defects as a major pathway for degraded blocking performance [47]; by stepwise temperature control that induced SAM self-healing, the Al_2_O_3_ inhibition window was extended to 60 cycles, about 6.6 nm. Studies combining nucleation-promoting molecules with selective resists support the same trend [48,49]. Recent SAM strategies are shifting from maximizing initial completeness alone to jointly tuning GA activation and NGA barrier recovery within each cycle.

Small-molecule inhibitors (SMIs) can be repeatedly supplied in the gas phase, making them easier to integrate into three-dimensional structures and cluster tools. Representative examples include the use of the Si precursor itself as an inhibitor [50], selective SiO_2_ deposition using an ABC-type cycle containing acetylacetone [51], and metal/dielectric-selective deposition of TiN using an aromatic inhibitor [52]. Subsequent work confirmed that the adsorbed structure of acetylacetone can be displaced by the precursor [53]. Even with the same inhibitor, blocking performance varied with the precursor ligand and the density of surface reaction sites [54,55,56,57,58]. The essential difference between SAMs and SMIs does not lie in the wet- versus vapor-phase distinction; rather, performance is governed by how long the inhibition layer survives during the ALD reaction and how easily the precursor can penetrate or displace it.

Recent SMI studies focus less on molecular footprint and more on time-dependent coverage and surface-specific adsorption orientation. Repeated adsorption and removal of 1-nitropropane, and sequential application of mutually orthogonal SAMs, showed that an inhibition layer can be reconstructed during processing [59,60]. Comparisons of inhibitors of different sizes showed that larger molecules do not always provide better blocking; the connectivity of gaps between molecules and their adsorption orientation govern precursor penetration [61]. A comparison of aniline, pyrrole, and pyridine likewise found that performance ranking is not explained by molecular planarity alone but depends on the oxidation state and bonding orientation of Cu/CuO_x_ [62]. Selecting an SMI therefore requires considering thermal stability, compatibility with the precursor, and post-process removability, in addition to selective adsorption and packing density [63]. In this sense, an SMI is less a scaled-down SAM than a dynamic reactive layer that forms, is damaged, and recovers with every pulse.

Spectroscopic and computational studies interpret this dynamic reactive layer at different time and length scales. Temperature-dependent vibrational spectroscopy tracked how local defects in alkanethiol SAMs lead to Al_2_O_3_ nucleation [47]. Density functional theory has distinguished possible reaction pathways for inhibitor adsorption, hydrolysis, ligand exchange, and inhibition-layer degradation [64]. Grand canonical Monte Carlo and random sequential adsorption models have quantified competitive precursor–inhibitor adsorption and maximum packing at finite coverage [65,66], and lattice-independent kinetic Monte Carlo simulations have shown that fluctuations in adsorption and desorption can create transient penetration pathways [67]. Experimental evidence that precursor miscibility within the inhibition layer promotes the loss of selectivity [68] is consistent with these computational results. The selectivity lifetime is thus more sensitive to the coupling of adsorption free energy, coverage fluctuations, and precursor–inhibitor miscibility than to the static size of the inhibitor molecule alone [69].

Beyond adsorption–desorption equilibrium and miscibility, the inhibitor layer itself is not chemically inert to the ALD half-reactions. In situ infrared spectroscopy of an ABC-type SiO_2_ process showed that the co-reactant plasma step does not fully remove adsorbed acetylacetone; the persisting species are fragments of the original inhibitor molecule, and these fragments hinder the readsorption of fresh inhibitor in the following cycle, so that selectivity degrades cumulatively even when each individual cycle appears well behaved [70]. Adding a dedicated fragment-removal step before inhibitor reapplication more than doubled the maximum selectively deposited thickness in that system, from about 1.6 nm to about 2.7 nm [70]. This fragmentation-and-reapplication pathway is conceptually distinct from the simple desorption and precursor-penetration pathways discussed above and indicates that inhibitor chemistry must be evaluated cycle-by-cycle rather than from a single steady-state snapshot. Whether organic species desorbed from the NGA can redeposit on, or otherwise poison, the GA—for example by co-adsorbing with the precursor and altering its reactivity—is a related question that has received comparatively little direct experimental attention in the AS-ALD literature to date, and remains an open point for future in situ studies that track both surfaces simultaneously.

The distinction between SAM- and SMI-based inhibition also becomes sharper away from idealized planar GA/NGA interfaces. On a planar coupon, a SAM benefits from a large, continuous area over which to self-organize into a densely packed layer, so its initial barrier quality is close to the thermodynamic optimum for that head–tail chemistry. At singular points of a real pattern—line edges, corners, and via bottoms—the same molecule has less lateral space in which to order into a close-packed arrangement, so the initial barrier is correspondingly less complete even before any ALD cycling begins. SMIs, because they are redosed every cycle rather than relying on a single close-packed assembly, are in principle less sensitive to this loss of ordering at edges, but they are more exposed to gas-phase transport limitations at singular points in three-dimensional structures, such as reduced local partial pressure or increased residence time relative to open, planar areas. Position-resolved studies on scaled Cu/SiO_2_ line patterns down to 10 nm pitch have shown that selectivity loss is not spatially uniform and is measurably worse near pattern edges than in the open-field regions of the same sample [71], consistent with this picture, though so far documented for a limited number of SAM chemistries; comparable edge-resolved data for SMI-based processes in three-dimensional structures remain scarce.

### 2.3. Pattern Topography and the Spatial Origin of Selectivity Breakdown

Even for the same material combination, selectivity varies with pattern topography. In nanopatterns, film grown in the GA can expand laterally and encroach on the NGA [72], and surface diffusion together with the pattern boundary has been reported to govern the increase in film width [28,29]. At edges and corners, the inhibition layer tends to form less completely than on flat regions, and because the precursor can approach from multiple directions, nucleation can begin there before it does in the interior of the NGA.

The first stable nucleus does not remain a local defect. Once formed, it presents a reaction surface similar to that of a conventional ALD film during subsequent cycles, and the rate of non-selective growth increases rapidly. Defect sites and pinholes in the inhibition layer, precursor penetration and inhibitor degradation, and lateral overgrowth appear to be distinct phenomena on the surface, but they converge on the same outcome in that each creates a new growth surface in the NGA.

Because of this spatial dependence, the average film thickness on a planar coupon is insufficient to judge selectivity. On patterned coupons, boundary expansion, corner nucleation, nucleus density in the NGA, and structure-to-structure variation become the key observables. To emulate device processing, verification must also include whether selectivity is maintained up to the target thickness under conditions where etch residues and plasma-damaged surfaces remain. Framing selectivity breakdown as a single transition event together with its spatial distribution allows inhibitor development and process-correction strategies to be compared on the same basis.

Isolating positional effects within a pattern requires an experimental design that can separately observe the top, sidewall, bottom, and boundary regions. Ensemble-type RBS analysis of multiple nanowire patterns simultaneously has quantified position-dependent areal density [18]. As shown by early microcontact-printing studies [39,40,41], spatial resolution is limited not only by inhibitor chemistry but also by the patterning method and the sharpness of the inhibition boundary. Molecular design and defect correction must therefore address the spatial stability of the boundary in addition to chemical selectivity.

A related and often underexamined variable is the processing history of the surface itself, independent of pattern geometry: whether the GA and NGA are analyzed as-deposited or after they have passed through integration steps such as etching, chemical-mechanical polishing (CMP), or wet/dry cleaning. In situ spectroscopic ellipsometry of octadecanethiol self-assembly on copper has shown that the extent of native-oxide growth—determined by how long, and by what method, the copper was cleaned before SAM deposition—directly changes the achievable inhibition-layer quality and, in turn, the nucleation delay obtained in a subsequent ALD process [73,74]. More broadly, copper surfaces treated with different acids or oxidizing agents prior to SAM formation differ systematically in composition and morphology, which alters how the inhibitor nucleates and packs on the metal [74]. Because CMP- and etch-exposed surfaces in real interconnect and memory structures are essentially never in the same state as a freshly deposited, unpatterned reference film, selectivity data obtained on pristine coupons should be regarded as an upper bound; the AS-ALD literature would benefit from more studies that deliberately compare identical inhibitor/precursor chemistries on as-deposited versus etch-, CMP-, or clean-exposed GA and NGA surfaces within the same pattern.

## 3. Strategies for Extending the Selectivity Window

Approaches for extending the selectivity window target different points along the pathway leading to stable-nucleus formation. Precursor–inhibitor combinations reduce initial adsorption and penetration in the NGA; inhibitor resupply restores barriers damaged during the reaction; ALD/ALE supercycles remove nuclei that have already formed before they can grow into a continuous film; and real-time metrology combined with reactor control detects the onset of selectivity loss and adjusts process variables accordingly. These approaches are complementary rather than mutually exclusive and are more effective when used together.

### 3.1. Molecular Compatibility Among Precursor, Inhibitor, and Substrate

A precursor for conventional ALD needs only sufficient volatility, thermal stability, fast surface reaction, and low impurity content. AS-ALD adds the further requirement of simultaneously satisfying reactivity in the GA and unreactivity in the NGA. Small precursors are favorable for diffusivity and growth rate but can more readily penetrate the inhibition layer. High Lewis acidity or reactivity may attack the head or tail functional groups of the inhibitor. Bulky ligands are advantageous for steric blocking but may slow nucleation in the GA and transport within high-aspect-ratio structures.

Experimental results show that no single precursor or inhibitor works universally across all substrates. With the same inhibitor, selectivity varied substantially with the choice of Al precursor [54,55], and a correlation between reaction-site density and precursor choice has also been confirmed [56]. In a study that varied both the inhibitor and the Al precursor together, adsorption orientation and precursor–inhibitor interaction proved to be more direct variables than molecular size [58,75]. Recent carbon-free inhibition layers and computation-guided design are moving toward reducing residual carbon while simultaneously predicting substrate bond energy, precursor penetration, and displacement reactions [76,77,78].

The goal of molecular design is not to select the least reactive precursor possible, but to find a combination in which the difference between the GA growth rate and the NGA nucleation probability is maintained up to the target thickness. Inhibitor–substrate bond energy and coverage, precursor adsorption–desorption kinetics, and stability toward the co-reactant and byproduct removal are not independent variables but are coupled to one another. Optimizing a single item can trigger a loss of selectivity at another stage.

Computational and data-driven methods are useful for narrowing a multivariable search space, but selectivity cannot be reliably predicted from a single descriptor. Machine learning using molecular descriptors can rapidly screen structural trends among candidate precursors [20], and first-principles calculations provide adsorption energies, ligand-exchange barriers, and inhibition-layer degradation pathways [64,66,69]. Grand canonical and kinetic Monte Carlo methods describe competitive adsorption and time-dependent formation of gaps at finite coverage [65,67], and reactor-scale computational fluid dynamics connects surface reactions to dosing, purge, pressure, and wafer position [19]. Linking a single calculated bond energy to an actual selectivity window and tool conditions requires hierarchical verification that ties together thermodynamics, coverage statistics, reaction kinetics, and reactor-scale transport. Beyond these physics-based and descriptor-based approaches, machine-learning methods are increasingly being applied directly to ALD and AS-ALD process data rather than only to precursor screening. A recent perspective surveys applications of artificial intelligence across ALD process optimization, in situ data analysis, and materials discovery, and identifies area-selective processes specifically as a setting where the combinatorial size of the precursor–inhibitor–substrate space makes data-driven search particularly attractive [79]. At the reactor level, a run-to-run controller that combines a multiscale kinetic Monte Carlo/machine-learning model with an exponentially weighted moving-average update rule has been used to keep the growth per cycle of a spatial AS-ALD reactor at a target setpoint despite pressure and kinetic disturbances, illustrating how the closed-loop philosophy discussed in Section 3.4 can be implemented with a learned process model in the loop [80]. These reactor-control applications remain at an earlier stage of maturity than computational approaches to inhibitor design, and closing the loop between molecular-level machine learning and reactor-level machine-learning control is still largely an open engineering problem.

### 3.2. Dynamic Maintenance of the Inhibition Layer and Reactor-Condition Control

It is unrealistic to assume that a SAM formed once remains undamaged through every ALD cycle. Sequential regeneration of SAMs has been shown to extend the selectivity window [81], and this has been followed by supercycles that repeatedly supply or selectively remove a vapor-phase inhibitor [82,83,84]. This approach does not rely solely on the completeness of the initial inhibition layer; because it periodically repairs desorption and chemical damage occurring during the reaction, it is advantageous for tool integration.

Reactor conditions deserve as much attention as molecular design. Increasing the precursor dose is favorable for GA saturation and coverage of high-aspect-ratio structures, but it also increases NGA physisorption, inhibitor displacement, and reactant residence time in the reactor. A longer purge can improve selectivity but reduces throughput. A study that used an N_2_ co-flow to reduce the residence time and interaction between TMA and a phosphonic-acid inhibitor improved selectivity through gas delivery alone, without introducing a new inhibitor [85].

Inhibitor resupply, precursor dose, carrier-gas flow, purge time, and pressure are not independent variables; together they determine the instantaneous coverage of the NGA surface and reactant residence time. In three-dimensional structures, the exposure history differs between the inlet and the deep interior, so optimal conditions found on a planar substrate do not guarantee the same selectivity throughout the entire interior.

Inherent-selectivity or activation-based processes that do not use an inhibitor widen the selectivity window by taking a different reaction pathway altogether. In ZnS ALD at 400–500 °C, film grew on Ti/TiO_2_ while no growth was detected on native SiO_2_ or Al_2_O_3_ over 250 cycles; at 450 °C, a selective film of about 80 nm formed on Ti/native-SiO_2_ patterns and about 23 nm on TiO_2_/Al_2_O_3_ patterns [86]. In a ZrO_2_ process, precursor selection alone amplified the growth difference between Si and Cu by more than a factor of ten [87]. A Hf_1-x_Zr_x_O_2_ process using catalytic O_2_ activation on Ru/TiN formed a selective film of about 7 nm and achieved device-quality properties after post-treatment [88]. A Ru process using the chemoselective adsorption of alkyl aldehyde on nitride surfaces directly controlled the reactive sites of the NGA [89]. A low-temperature Co process based on an OTS-SAM and a SiO2 process combining a catalytic growth surface with phosphonic-acid passivation are examples of achieving selectivity within the thermal budget of BEOL [90,91]. The advantage of these approaches lies not simply in eliminating an auxiliary molecule, but in the ability to choose whichever pathway—precursor reaction rate, catalytic activation, or surface passivation—maximizes the difference between GA and NGA.

### 3.3. Defect Correction Using ALD/ALE Supercycles

If nucleation in the NGA cannot be completely suppressed, it is more practical to remove the nuclei before they grow into a continuous film. An ALD/ALE supercycle composes deposition and etching as separate self-limiting reactions, maintaining net growth in the GA while removing initial nuclei in the NGA. A process combining thermal ALD and thermal ALE extended the selectivity window [92], and a study on MoS2 used inherent selectivity together with ALE correction to reduce NGA residues [93].

In this process, the balance among the GA deposition amount per cycle, the NGA nucleus-removal amount, and the GA loss amount is critical. Insufficient etching allows NGA nuclei to accumulate, while excessive etching damages the GA film thickness and interface quality. The thickness balance for one supercycle can be expressed by Equations (2) and (3).

*Δt_GA_* = *g_GA_* − *e_GA_* > 0 (2)

*Δt_NGA_* = *g_NGA_* − *e_NGA_* ≤ 0 (3)

**Figure 3:**
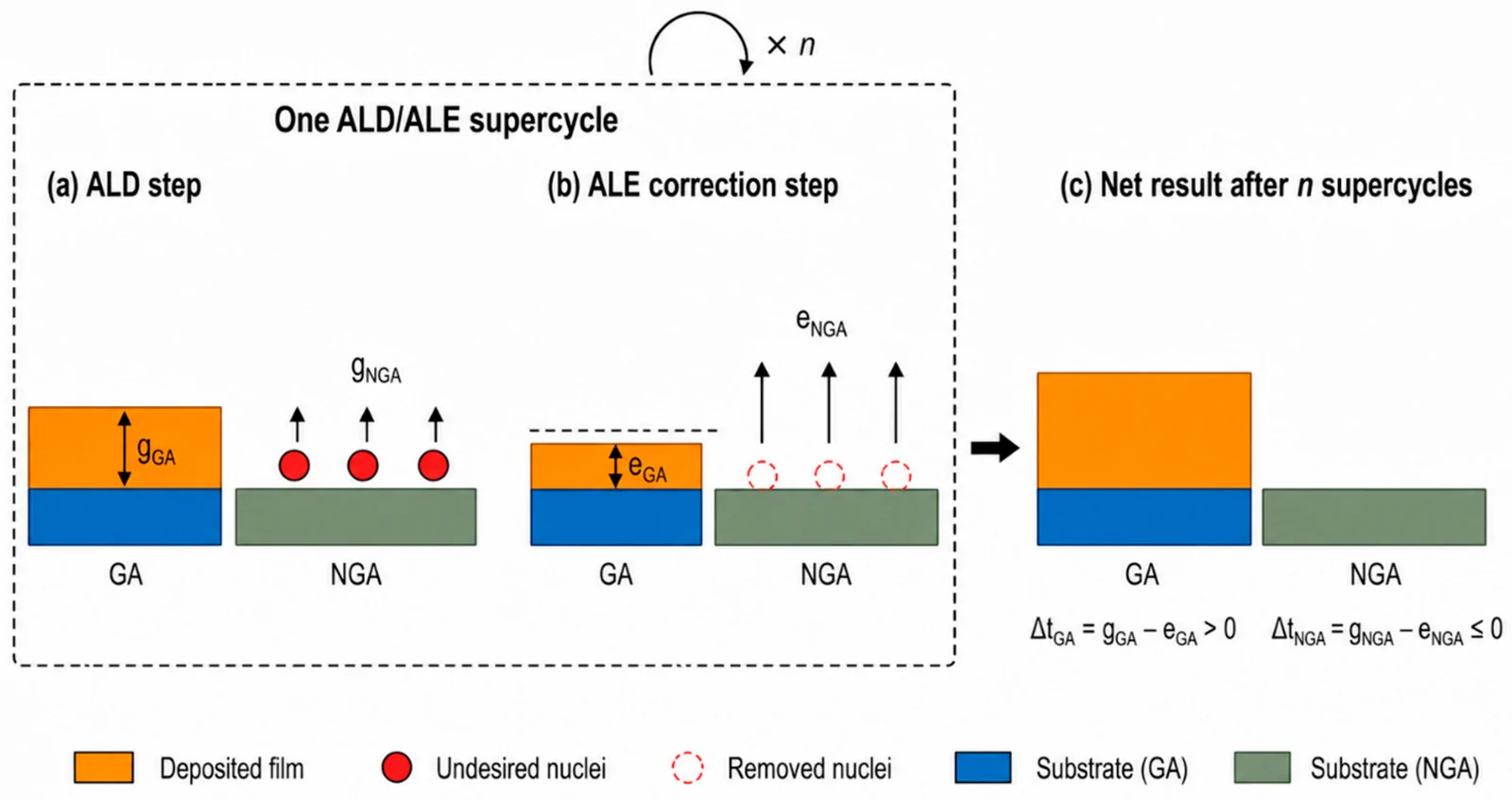
Concept of defect correction using an ALD/ALE supercycle. (**a**) During the ALD step, a film grows in the GA while an initial nucleus may form in the NGA. (**b**) During the ALE correction step, the NGA nucleus is removed while GA loss is minimized. (**c**) After n repetitions, the GA maintains net growth (Δt_GA_ = g_GA_ − e_GA_ > 0), while the NGA net growth is suppressed to zero or below (Δt_NGA_ = g_NGA_ − e_NGA_ ≤ 0).

In Equations (2) and (3), g and e denote, respectively, the thickness increase during the deposition step and the amount removed during the etching step. Under ideal correction conditions, net growth in the GA remains positive while net growth in the NGA is at or below zero. In practice, the reactant dose and nucleus size during the deposition step alter the subsequent etch rate, so optimizing ALD and ALE as independent recipes is insufficient [92,94]. A window must be found in which NGA nuclei are removed before converting into a continuous film while the GA film continues to accumulate toward the target thickness. This balance is illustrated schematically in Figure 3.

Combining ALD and ALE improves selectivity at the cost of increased process time, tool compatibility requirements, and byproduct-removal burden. Rather than applying it uniformly to every AS-ALD process, it is more reasonable to apply it preferentially to material combinations for which the target thickness is difficult to achieve with an inhibitor alone and for which a sufficient difference exists between the GA and NGA etch reactions.

Defect correction has been implemented through selective removal of initial nuclei, deposition-etch supercycles, and substrate-dependent etch reactions. In area-selective molecular layer deposition, defects were removed before they could form a continuous film, restoring selectivity [95]. Studies employing selective etching after Ru ALD, and alternating deposition-etch processes, periodically removed NGA deposits while maintaining net GA growth [96,97]; the relative rate of each step jointly determined the selectivity window and the extent of GA loss. In a study on HfO2, even when the deposition-step selectivity itself was not large, differences in film structure and ALE rate between Co and SiO2 were exploited to obtain a final positional selectivity greater than 4 nm [98]. The design variables of defect correction thus extend beyond nucleus size to include crystallinity, interfacial bonding, and etch-stop behavior.

### 3.4. Early Detection of Selectivity Loss and Manufacturing Process Control

Anomalies in AS-ALD first appear as changes in the small population of NGA nuclei or in the inhibitor bonding state, before the average film thickness itself changes. Quartz crystal microbalance and spectroscopic ellipsometry are useful for tracking average growth behavior but are insufficient on their own to detect early, localized nucleation. Surface bonding states can be tracked by in situ IR or RAIRS, the onset of nucleation by optical signals, and reaction byproducts by exhaust-gas spectroscopy. Combining post-process AFM, SEM, and XPS analysis can compensate for the differing detection limits of each technique.

In manufacturing, selectivity value alone is not the only concern. Within-wafer uniformity and defect density, inhibitor residue and chamber memory effects, precursor cross-contamination and process reproducibility, and throughput are all part of the evaluation. On 300 mm wafers and high-aspect-ratio structures, position-dependent dosing and purge differences can appear directly as a spatial distribution of selectivity. Process control is thus better approached as closed-loop operation—adjusting dose, purge, inhibitor resupply, and correction cycles in response to early anomaly signals—rather than as a search for a single end point.

Few examples of real-time correction systems have so far been sufficiently demonstrated at a manufacturing scale. Nonetheless, moving away from analyzing selectivity loss only after the process and toward detecting and correcting the onset of nucleation during the process is necessary for laboratory-scale selective deposition to become a reproducible manufacturing process.

For plasma-assisted AS-ALD and ALE, separately controlling radical flux and ion energy is particularly important. Plasma-assisted ALD offers high reactivity even at low temperature, but radical recombination and directional ion bombardment can cause the reaction to vary with depth in high-aspect-ratio structures [99]. Industrial ALE studies note that even when self-limiting adsorption and activation are separated, ion energy distribution, surface charging, byproduct desorption, and chamber-wall condition can alter the etch amount per cycle and the selectivity [100,101]. Recent studies on plasma ALE and atomic-level plasma processing have summarized how ion-radical synergy enables atomic-layer control while simultaneously introducing damage and directionality as additional variables [102,103]. The observation target for closed-loop control is therefore not a single variable such as precursor dose, but the entire process state, including plasma-source condition and the position-dependent ion/radical exposure across the wafer.

## 4. Semiconductor Process Applications and Level of Demonstration

The application value of AS-ALD is difficult to judge from whether selective deposition has been demonstrated alone. What matters is which device-level function the process replaces or complements, and whether that effect translates into electrical performance and reliability. The following subsections classify the level of demonstration into device-like nanopatterns, electrical devices, and material/concept stages, and review the applicability and manufacturing readiness of AS-ALD for back-end-of-line interconnects, memory, and transistors.

### 4.1. Self-Aligned Patterning and BEOL Interconnects

The area in which AS-ALD is most directly connected to semiconductor manufacturing is self-aligned patterning in back-end-of-line (BEOL) interconnects. As wiring and vias shrink, the fraction occupied by barrier and liner films increases, and overlay error can lead to shorts or leakage between adjacent lines. Here, AS-ALD, rather than covering the entire structure, forms a cap only on metal lines or grows an etch-stop or insulating film only on the surrounding dielectric, widening the alignment margin for subsequent via processes [5,6,7,104].

**Figure 4:**
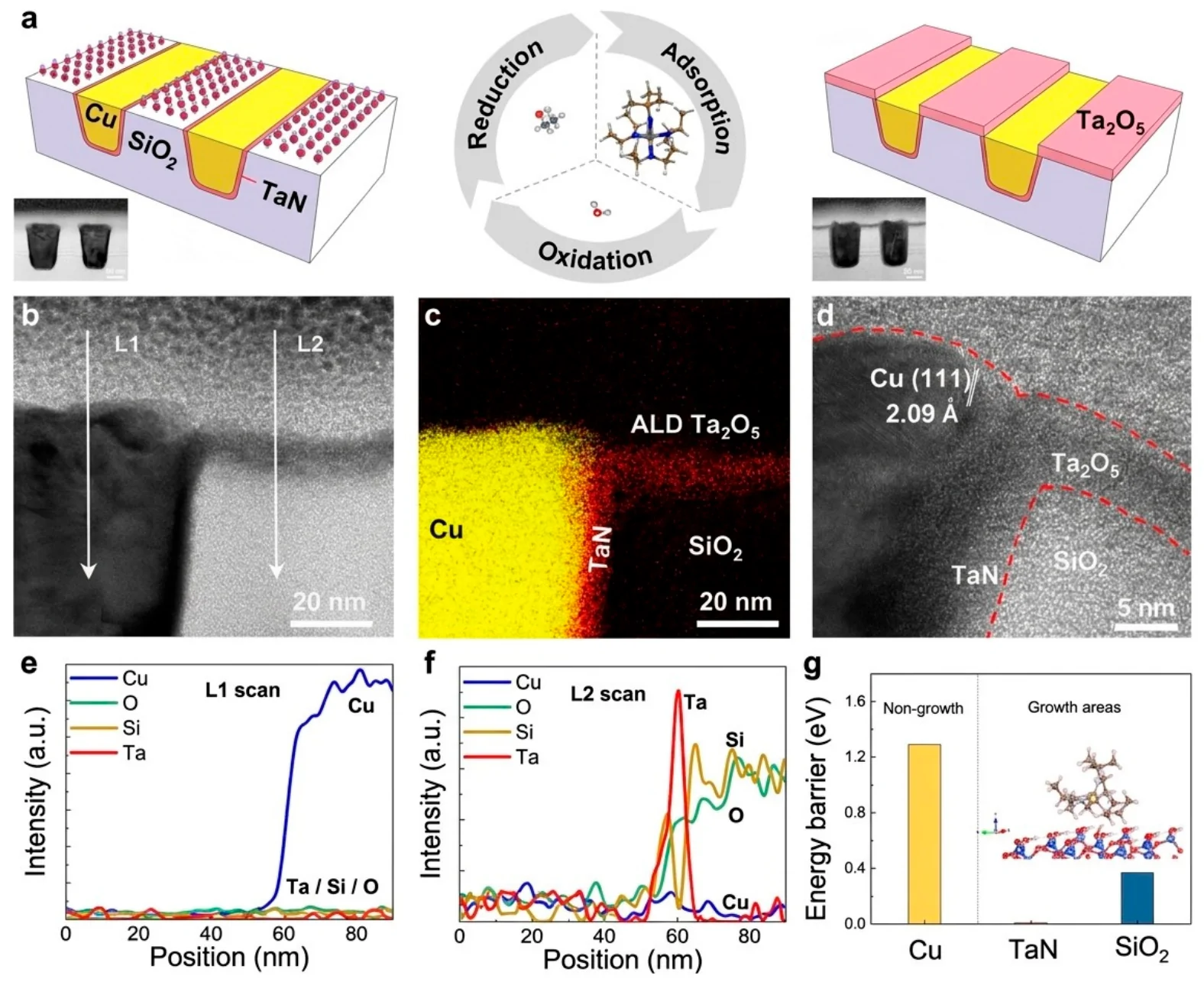
Self-aligned TaOx formed by redox-coupled inherently selective ALD on a Cu/SiO_2_ nanopattern. (**a**) Process concept and pattern structure; (**b**–**d**) cross-sectional TEM images and elemental distribution; (**e**,**f**) elemental line profiles across the Cu and SiO_2_ regions; (**g**) comparison of precursor reaction barriers on Cu, TaN, and SiO2. Adapted and reconstructed from Li et al. [22], Figure 4 (CC BY 4.0).

Studies using metal/dielectric patterns share the common feature of converting an existing boundary into a self-alignment reference, even when the materials differ. An amino-functionalized alkoxy-silane passivation layer limited Ru growth on nanometer-scale Cu lines [105]. A study aiming at fully self-aligned vias controlled the growth location of AlO_x_ and Al-silicate on Cu/SiO_2_ [104]. A study that selectively deposited Ta oxide via a redox-coupled surface reaction achieved the same boundary function without an organic inhibitor [22]. What matters for BEOL is less the identity of a particular film than the function of using the metal/dielectric boundary to align a cap, etch-stop, or insulating boundary without an additional overlay step. The implementation results on Cu/SiO_2_ nanopatterns are shown in Figure 4.

However, confirming selective deposition alone does not establish BEOL applicability. Inhibitor residue can affect metal contact resistance and cap adhesion, and plasma or chemical pretreatment can alter the composition and reliability of low-k dielectrics. Device-level evaluation cannot stop at selectivity and pattern-shape preservation; contact resistance and electromigration, time-dependent dielectric breakdown, via-chain yield, and compatibility with subsequent cleaning and etching must also be examined. The current literature is comparatively rich at the nanopattern level but limited in cases that report electrical characteristics and long-term reliability.

BEOL applications are more usefully grouped by process function than by deposited material. For selective metal formation, a low-temperature Co process using an OTS-SAM achieved positional control of metal/dielectric patterns within a limited thermal budget [90]. For selective dielectric formation, an SiO_2_ process combining catalytic surface reaction with phosphonic-acid passivation reversed the GA/NGA roles of metal and dielectric [91]. Aiming at a BEOL-compatible channel material, an SnS_2_ process used SiO_2_ as the GA and Al_2_O_3_ as the NGA, forming a selective film of about 7 nm using an acetylacetone-injection/SnS ALD/H_2_S-plasma supercycle [106]; here, acetylacetone not only suppressed nucleation but also protected the NGA Al_2_O_3_ from the H_2_S plasma. The BEOL-integrable window is determined not only by low-temperature selectivity but also by the range over which the inhibition layer can withstand plasma exposure and subsequent regeneration.

### 4.2. High-Aspect-Ratio Memory Structures and Selective Thin-Film Formation

In DRAM capacitors, ALD is used to uniformly form a high-k dielectric across the entire depth of a deep electrode structure. When the primary requirement is step coverage, blanket ALD remains the default process, and AS-ALD does not replace it. Where AS-ALD can contribute is in adding a thin dielectric only to a specific electrode or interfacial layer, or in locally tailoring interfacial structure by suppressing nucleation on one of two different oxide surfaces. A study that selectively fluorinated a single ZrO_2_ surface and then controlled Al_2_O_3_ growth demonstrated this possibility [12]. Direct evidence confirming capacitance, leakage, and endurance in an actual DRAM capacitor, however, is not yet available.

The tunnel oxide, charge-trap layer, blocking oxide, and gate-related films of 3D NAND likewise must uniformly cover the entire channel hole or gate trench, so conventional ALD is applied first. The distinctiveness of AS-ALD lies in bottom-up gap-fill that must grow only at specific material boundaries during gate-replacement processes, at word-line insulation, or at selective spacers and passivation—that is, only on part of the top, sidewall, or bottom [5,6,7,107]. A high aspect ratio alone does not necessitate AS-ALD; the advantage of selective deposition arises only when different surfaces within one structure must be distinguished.

Studies that measure, in structures reflecting actual stack count and aspect ratio, the depth-dependent concentration of inhibitor and precursor, purge efficiency, the selectivity window, and boundary expansion together are rare. Reports linked to channel current, word-line leakage, dielectric breakdown, and retention characteristics are likewise insufficient. Based on the current level of evidence, DRAM and 3D NAND remain closer to the stage where application sites have been identified conceptually; extrapolating selectivity obtained on planar substrates or simple trenches directly to memory-device demonstration is difficult.

This scarcity is, in part, a metrology problem rather than only a materials problem. For buried, high-aspect-ratio stacks such as 3D NAND channel holes and DRAM deep capacitors, cross-sectional transmission electron microscopy (TEM) combined with focused-ion-beam sectioning remains the technique that can most directly resolve local film thickness and boundary sharpness at the relevant depths, since optical and X-ray-based critical-dimension metrology cannot directly access buried critical dimensions at these aspect ratios [108]. TEM-based inspection is inherently destructive, low-throughput, and samples only a small number of sites per wafer, so it is poorly suited on its own to the statistics needed to establish a selectivity window with confidence. Electrical measurements (capacitance, leakage, channel current) can in principle substitute for direct imaging, but they remain indirect with respect to selective growth itself; an electrical signature depends on the full integration flow, so a change in device performance cannot be unambiguously attributed to the AS-ALD step in isolation without a matched, imaging-based reference. Progress on DRAM- and 3D-NAND-relevant AS-ALD will likely require pairing higher-throughput indirect metrology, such as in-line electrical test structures, with periodic, targeted TEM verification, rather than relying on either approach alone.

In high-aspect-ratio structures, selectivity can appear less as a growth/non-growth dichotomy and more as a depth-dependent growth-rate gradient. Bottom-up gap-fill using a graded surface deliberately varied nucleation or growth rate between the bottom and the inlet of the structure to suppress void formation [109]. This work shows that uniform inhibitor delivery is not the only possible goal; depending on the process purpose, depth-dependent non-uniformity can itself be exploited for gap-fill or insulation function. However, the effect of such gradients on wafer-to-wafer reproducibility and electrical uniformity requires separate verification.

### 4.3. Transistors and Next-Generation Memory Devices

In gate-all-around field-effect transistors (GAA-FETs) and nanosheet transistors, positional control between adjacent materials—the high-k/metal gate stack, inner spacers, dielectrics adjacent to source/drain, and contact insulation—is important. Conventional ALD is suitable for the uniform deposition of the gate dielectric wrapping the nanosheet. Forming a dielectric or cap only on a specific sidewall or around the source/drain, however, requires subsequent etching. AS-ALD has been proposed as a means of forming local spacers and dielectrics and of self-aligning metal/dielectric boundaries [5,6,7,107], but direct demonstrations of performance improvement in a completed GAA-FET remain limited.

**Figure 5:**
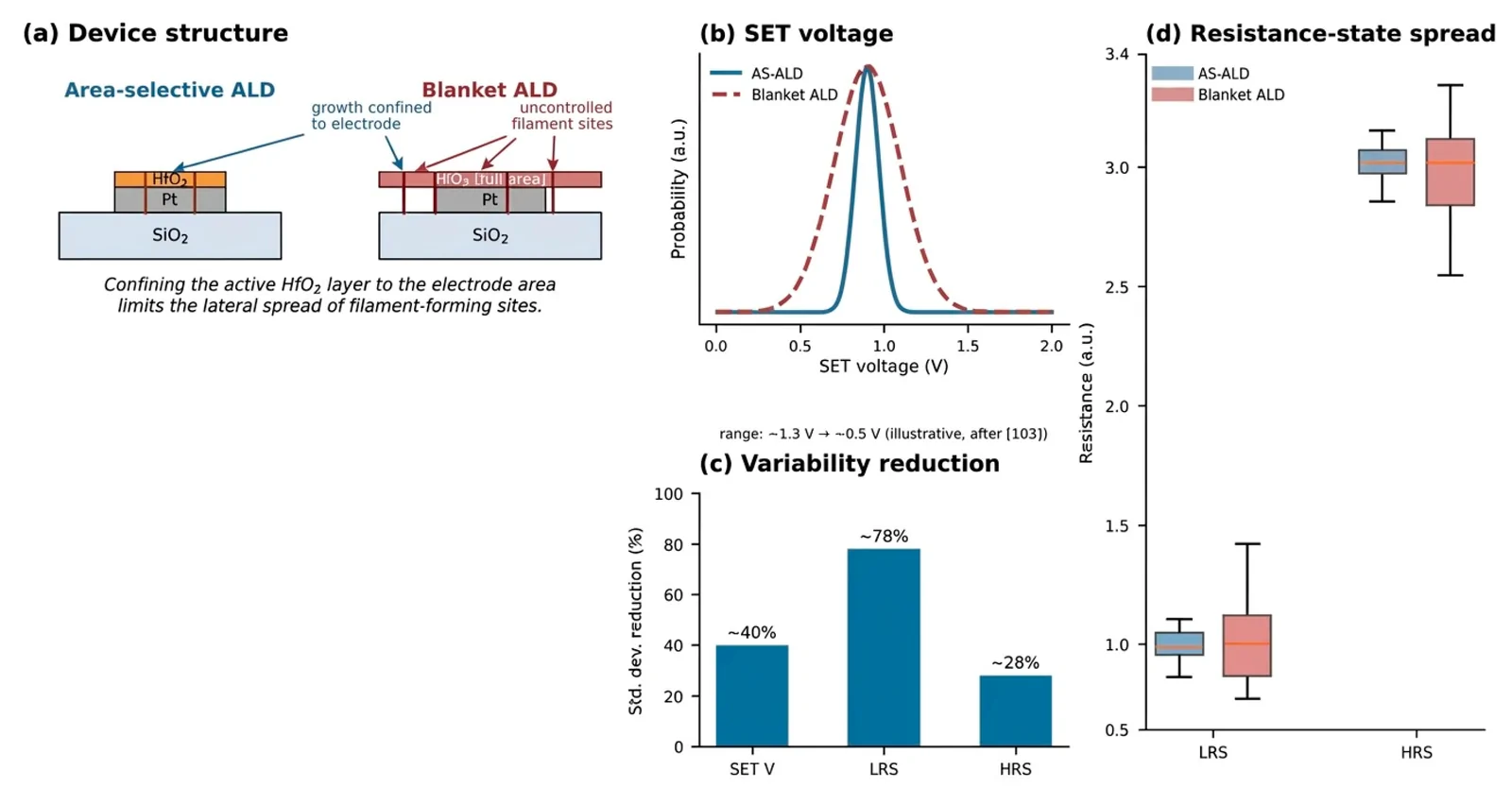
Schematic illustration of selective HfO_2_ deposition and its effect on RRAM electrical variability. (**a**) Device cross-sections comparing an area-selectively deposited HfO_2_ cap confined to the Pt electrode with a blanket-ALD HfO_2_ film covering the full Pt/SiO_2_ pattern; (**b**) representative SET-voltage distributions; (**c**) illustrative reduction in the standard deviation of SET voltage, low-resistance-state (LRS), and high-resistance-state (HRS) values; (**d**) schematic spread of the LRS and HRS distributions. Panels are original schematic illustrations drawn by the authors to summarize the trends reported by Oh et al. [110] and do not reproduce the original figures or data [103].

In resistive random-access memory (RRAM), device-level effects have been reported. Oh et al. fabricated a crossbar RRAM by selectively depositing about 5 nm of HfO_2_ onto the Pt electrode of a Pt/SiO2 pattern [110]. Compared with a blanket-ALD device, the SET-voltage distribution range of the AS-ALD device narrowed from about 1.3 V to 0.5 V, and the standard deviation of the SET voltage decreased by about 40%. The standard deviations of the low-resistance and high-resistance states also decreased by about 78% and 28%, respectively. This case demonstrates, through electrical results, that confining the growth area of the active dielectric to the electrode can reduce filament-location and device-to-device variability. A comparison of the selective-deposition location and device variability is shown in Figure 5.

The contrast between RRAM and GAA-FET clarifies why applicability and demonstration level must be distinguished. In RRAM, selective deposition led directly to a change in electrical characteristics. GAA-FET applicability, by contrast, is more often inferred from thin-film or patterned-substrate results. In the front-end-of-line, inhibitor residue and fluorination, plasma damage, and carbon contamination can alter interface traps, fixed charge, threshold voltage, and mobility. Transistor applicability therefore cannot be judged from high selectivity alone; C-V characteristics, leakage, bias-temperature instability, and mobility must subsequently be evaluated on a MOS capacitor or transistor.

Examples involving next-generation channel and high-k materials show that selective deposition and device-grade film quality are separate issues. A Hf_1-x_Zr_x_O_2_ process limited the growth location through catalytic O_2_ activation on Ru/TiN and secured antiferroelectric properties after post-treatment [88]. An SnS_2_ nanosheet process targeted a BEOL-compatible transistor channel, mitigating H2S-plasma-induced NGA damage through the protective function of the inhibition layer [106]. Selectivity, composition, crystallinity, and charge transport cannot be evaluated independently; selective deposition is meaningful only when the electrical quality of the final channel or gate stack is preserved.

### 4.4. Process-Integration Challenges Limiting Device Application

The largest gap in device application lies between the initial selectivity measured on planar coupons and the process performance required by actual structures. Patterned surfaces retain etch residues, line-edge roughness, corners, mixed termination states, and plasma-damaged sites. These local regions become the pathway along which a stable nucleus first forms in the NGA. Average thickness on a full-area substrate is insufficient to judge device applicability; nucleus density at pattern boundaries, lateral overgrowth, and defect density at the target thickness must also be confirmed.

In three-dimensional structures, not only surface composition but also topography and angle of incidence alter selectivity. Topographically selective deposition has been proposed as an approach that distinguishes the top, sidewall, and bottom of a structure made of the same material, and selective deposition inside trenches using ion-radical synergy or an amorphous-carbon inhibition layer has been reported [14,111]. However, as ion directionality, aspect ratio, shadowing effects, and inhibitor transport change, selectivity also varies with depth within the structure—a reason why mass transport must be verified separately from planar surface chemistry.

Process integration cannot be judged from a single selectivity value. It is jointly determined by the target film thickness, the tolerable boundary expansion and defect density, the thermal budget, inhibitor removability, compatibility with subsequent etching and cleaning, and electrical reliability. The current literature spans a wide range of results, from positional control in nanopatterns to the electrical characteristics of some devices, but the depth of verification differs greatly across materials and structures. Reporting substrate topography, target thickness, and level of electrical evaluation together provides a basis for comparing process maturity across the literature.

Extending the detection-limit discussion of Section 3.4 to device verification requires a staged evaluation framework. On planar coupons, adsorption and initial nucleation are analyzed [17,18]; on chemically patterned coupons, boundary shape and local defects are measured; in three-dimensional structures, position-dependent thickness and inhibitor coverage are confirmed [14,109,111]; and in final devices, resistance, leakage, variability, and reliability are evaluated. Because each stage reveals a different failure mechanism, the result at one stage cannot substitute for another. This verification flow constitutes a minimum roadmap for the transition to a manufacturing process.

## 5. Challenges and Future Directions for Process Integration

The next stage of AS-ALD research should not stop at accumulating new inhibitor or precursor examples. It is necessary to connect the pathway by which a stable nucleus forms in the NGA to surface reactions and tool variables. Precursor chemisorption and physisorption, desorption and surface diffusion, dissolution and penetration within the inhibition layer, and mass transport at pattern boundaries should be treated as parts of a single nucleation pathway, allowing results from different processes to be compared within the same framework.

The problem of imparting selectivity to chemically similar surfaces, or within a single material, also remains. Studies on oxide/oxide SAMs, local fluorination of a single oxide, and SiO_2_ plasma passivation have shown that nucleation can be differentiated by surface termination and pretreatment history even when bulk composition is identical or similar [12,13,42]. Surface analysis immediately after pretreatment is not sufficient; verification must extend to whether passivation is maintained over subsequent cycles, whether residues affect electrical characteristics, and whether spatial resolution is preserved on actual patterns.

From a process-integration perspective, it is more realistic to slow the loss of selectivity and remove nuclei early, rather than to aim for a perfectly clean initial NGA. Inhibitor regeneration and vapor-phase resupply, control of carrier gas and residence time, selective inhibitor removal, and ALD/ALE supercycles differ in implementation but share the common principle of periodically lowering the reaction probability in the NGA [81,82,83,84,85,92]. Performance should not be evaluated by selectivity improvement alone; net growth rate, process time, byproduct removal, chamber memory effects, and GA film damage must also be considered together.

Transitioning to a manufacturing process means connecting, under the same process conditions, position-dependent variation across 300 mm wafers, depth-dependent reactant transport in high-aspect-ratio structures, defect statistics in sub-10 nm patterns, compatibility with subsequent cleaning and etching, and electrical reliability. Wafer maps, defect distributions, and lot-to-lot variation more directly indicate process maturity than an average selectivity value. Once such data accumulate, AS-ALD can be evaluated not as an isolated example of selective deposition but as a reproducible self-aligned manufacturing process.

Research designs that connect computation, metrology, and process experiments are also important. Density functional theory and first-principles calculations propose candidate reaction pathways [64,66,69], and grand canonical and kinetic Monte Carlo methods predict competitive adsorption and defect-formation probability at finite coverage [65,67]. Computational fluid dynamics estimates position-dependent dosing and purge variation across a wafer [19]. These computational results should be validated against position-resolved areal density from RBS and nanoscale microscopy [18], bonding states observed by in situ spectroscopy, and electrical data from devices. Once common reporting items that reflect the industrial constraints of ALE and plasma processes are established [99,100,101,102,103], the literature can be compared based on the selectivity window and reproducibility rather than on the highest selectivity obtained under a specific condition. When verification linking multiple length scales is established, the accumulation of individual cases can be converted into genuine design rules.

## 6. Conclusions

AS-ALD adds positional selectivity to the atomic-scale thickness control and step coverage of ALD, enabling self-aligned manufacturing. However, the fact that no growth is detected in the NGA during the first several cycles is not, by itself, sufficient to judge practical utility. The success of an actual process depends on how long stable-nucleus formation can be delayed on device-like patterns and at the target thickness, how early any nucleus that does form can be removed, and how reproducibly this can be achieved across an entire wafer.

This review has not treated selectivity loss as a mere list of defects, inhibitor arrangement, precursor penetration, and thermal degradation. These phenomena all converge on a single transition event—the formation of a stable nucleus in the NGA. SAMs and SMIs delay this moment; combined precursor–inhibitor design reduces displacement and penetration; ALD/ALE limits the growth of nuclei that have already formed; and reactant dose, purge, co-flow, and plasma treatment together regulate molecular residence time and surface termination. Future progress will depend less on the performance of a single inhibitor or precursor than on integrating nucleation suppression, defect correction, and real-time metrology into one coherent process framework.

The level of demonstration differs markedly across application areas. For BEOL self-aligned vias and metal/dielectric patterns, nanopattern-level results have accumulated relatively well. In RRAM, confining the HfO2 active layer to a defined area translated into a device-level effect that reduced variation in SET voltage and resistance states. DRAM, 3D NAND, and GAA-FETs, by contrast, have identified plausible application sites and expected functions, but data on selectivity maintenance and electrical reliability in actual structures remain insufficient. Future work should specify, for each target film and GA/NGA combination, the required thickness, tolerable defect density, and the impact of subsequent processing.

Manufacturing implementation of AS-ALD is not simply a matter of achieving one high selectivity value. The thickness over which selectivity is maintained, preservation of pattern shape, defect density, electrical performance, and wafer-level reproducibility must all be satisfied together. The key criterion is not the initial selectivity S but the selectivity window that meets the target thickness and tolerable defect level for each specific device. When this criterion is reproduced across patterned and high-aspect-ratio structures at wafer scale, and when inhibitor chemistry and precursor design are connected with ALD/ALE and real-time metrology together with reactor control, AS-ALD can establish itself as a practical atomic-layer process for bottom-up, self-aligned manufacturing.

## Data Availability

No new data were created in this study. Data sharing is not applicable to this article.
